# Electrical Properties of Inorganic Perovskites CsPbBr_3_ Nanosheet Based on Strain Engineering

**DOI:** 10.3390/nano16201284

**Published:** 2026-10-10

**Authors:** Yi Wu, Ruixue Wang, Hui Dong

**Affiliations:** State Key Laboratory of Precision Electronic Manufacturing Technology and Equipment, School of Electromechanical Engineering, Guangdong University of Technology, Guangzhou 510006, China; wuyi@gdut.edu.cn (Y.W.); 2112401192@mail2.gdut.edu.cn (R.W.)

**Keywords:** strain engineering, CsPbBr_3_ nanosheets, schottky barrier, electrical properties

## Abstract

Fully inorganic CsPbX_3_ perovskites have emerged as promising optoelectronic materials because they retain the excellent optoelectronic properties of their hybrid counterparts while offering improved resistance to moisture and heat, although challenges such as soft crystal lattices, high ionic mobility, and easy defect formation remain. Strain engineering provides a novel approach to optimizing the optoelectronic properties of perovskite thin-film devices. This study demonstrates solution-grown CsPbBr_3_ nanosheets (12–22 nm thick, ~10 μm lateral size) on ITO substrates. The I-V characteristics of vertical tip/CsPbBr_3_/ITO structures were measured under varying compressive loads using conductive AFM, revealing load-dependent behavior. Analysis through thermionic emission modeling demonstrated an inverse correlation between the applied load and Schottky barrier height at the tip–sample interface. Furthermore, piezoresponse force microscopy quantifies thickness-dependent effective piezoelectric coefficients ($d_{33}^{eff}$ = 5.8, 3.7, and 1.2 pm/V for 12, 17.5, and 22 nm nanosheets). Consistent with piezoelectric electronic theory, these results demonstrate piezoelectric-effect-induced Fermi level lowering and consequent Schottky barrier reduction. These findings provide fundamental insights for strain-engineered perovskite optoelectronics.

## 1. Introduction

The growing demand for clean and renewable energy has made photovoltaics an urgent priority, and engineered nanostructures have emerged as promising candidates for high-performance solar cells, owing to their unique optical, electronic, and structural properties. For instance, III-V semiconductor nanowire arrays offer strong light absorption and efficient wave guidance [1], while core–shell nanowire heterostructures possess well-defined band-edge discontinuities that govern carrier transport [2]. These systems provide useful alternatives and a benchmark for evaluating emerging photovoltaic materials. Metal halide perovskites have recently attracted attention as important materials for their potential ability as solar cells, light-emitting devices, and photonic applications. Among the vast family of functional materials, perovskites represent a distinctive class of ionic compounds characterized by the general formula ABX_3_ [3], where A and B are cationic sites and X is an anionic site. When the A-site cation is an organic group (e.g., ${\mathrm{CH}}_{3}{NH}_{3}^{+}({\mathrm{MA}}^{+})$, $\mathrm{CH}{\left({\mathrm{NH}}_{2}\right)}_{2}^{+}({\mathrm{FA}}^{+})$), the material is classified as an organic–inorganic hybrid perovskite. Over the past two decades, extensive research on perovskite materials has revealed their exceptional optoelectronic properties, including high light absorption [4], high charge-carrier mobility [5], long carrier lifetimes [6] and tunable bandgaps [7]. These properties have enabled their widespread use in optoelectronic devices such as solar cells [8], light-emitting diodes [9], piezoelectric nanogenerators [10] and photo-detectors [11]. However, the ionic nature of perovskites also renders them susceptible to hydrothermal instability, posing a significant barrier to their large-scale industrial applications.

To address the stability issues of organic–inorganic hybrid perovskite materials, researchers have explored doping inorganic cesium ions (Cs^+^) into hybrid perovskites [12,13]. The fully inorganic cesium–lead perovskite materials (CsPbX_3_, X = Cl, Br, I) exhibit improved hydrothermal stability relative to their hybrid counterparts, although they still suffer from soft lattices, ionic migration, and defect formation [14]. Moreover, they demonstrate higher luminescence efficiency and quantum yield [7,15], making them promising semiconductor materials. Despite the outstanding electronic and optoelectronic performance of perovskite-based devices, challenges such as low switching ratios and mobility persist due to the ion-shielding effect, limiting their efficiency in optoelectronic device applications. Recently, strain engineering has emerged as a novel approach to modulate the optoelectronic properties of perovskite materials and enhance device stability. Xu [16] et al. constructed a WS_2_/CsPbBr_3_ van der Waals heterostructure planar photodetector on a flexible substrate, demonstrating a strain-dependent photoresponse. As the applied strain increased from −0.11% to 0.11%, the photoresponse rose from 0.1 A/W to 0.7 A/W, accompanied by an increase in photocurrent from 0.01 μA to 0.1 μA. Li [17] et al. investigated the mechanical properties of single-crystal CsPbBr_3_ grown by vapor–liquid–solid based on an in-situ scanning electron microscope (SEM) and explored the strain magnitude and distribution of the deformed CsPbBr_3_ by using finite-element simulations, in which the maximum local tensile elasticity could be as high as 4–5.11%. Additionally, flexible CsPbBr_3_ nanowire-based photodetectors exhibited excellent mechanical reliability, with nearly unchanged time–current profiles after 5000 bending cycles. These findings highlight strain engineering as a viable strategy for optimizing the optoelectronic performance of perovskite material devices.

Although strain engineering has been widely explored in flexible perovskite devices, the strain-dependent modulation of carrier transport in vertical perovskite junctions, particularly the quantitative relationship between local compressive strain, thickness-dependent piezoelectricity, and Schottky barrier height, remains largely unexplored. In this work, we investigated the influence of strain on electron transport in vertical metal/CsPbBr_3_ nanosheet/metal structures. CsPbBr_3_ nanosheets were grown on indium tin oxide (ITO) substrates by low-temperature liquid-phase synthesis. Different loads were applied to nanosheets of varying thicknesses using a conductive atomic force microscopy (AFM) tip, and the current–voltage (I-V) characteristics of the perpendicular tip/CsPbBr_3_/ITO structure were also systematically measured. The structure exhibited rectifying I-V characteristics and produced a clear correlation with strain. Combined with the thermal electron transport model, we extracted the Schottky barriers at the tip–sample interface under different strains. The result demonstrated that the enhancement of transport properties is due to the strain-induced reduction of the Schottky barriers. Unlike previous studies based on global bending strain in flexible devices, this work employs C-AFM tip-induced local compressive strain to quantitatively modulate the Schottky barrier and establishes a direct correlation between thickness-dependent piezoelectric coefficients and barrier lowering. These findings provide critical insights into strain-modulated carrier transport in inorganic perovskite materials, offering both technical and theoretical foundations for advancing their applications in optoelectronic devices.

## 2. Materials and Methods

### 2.1. Sample Preparation

**Figure 1:**
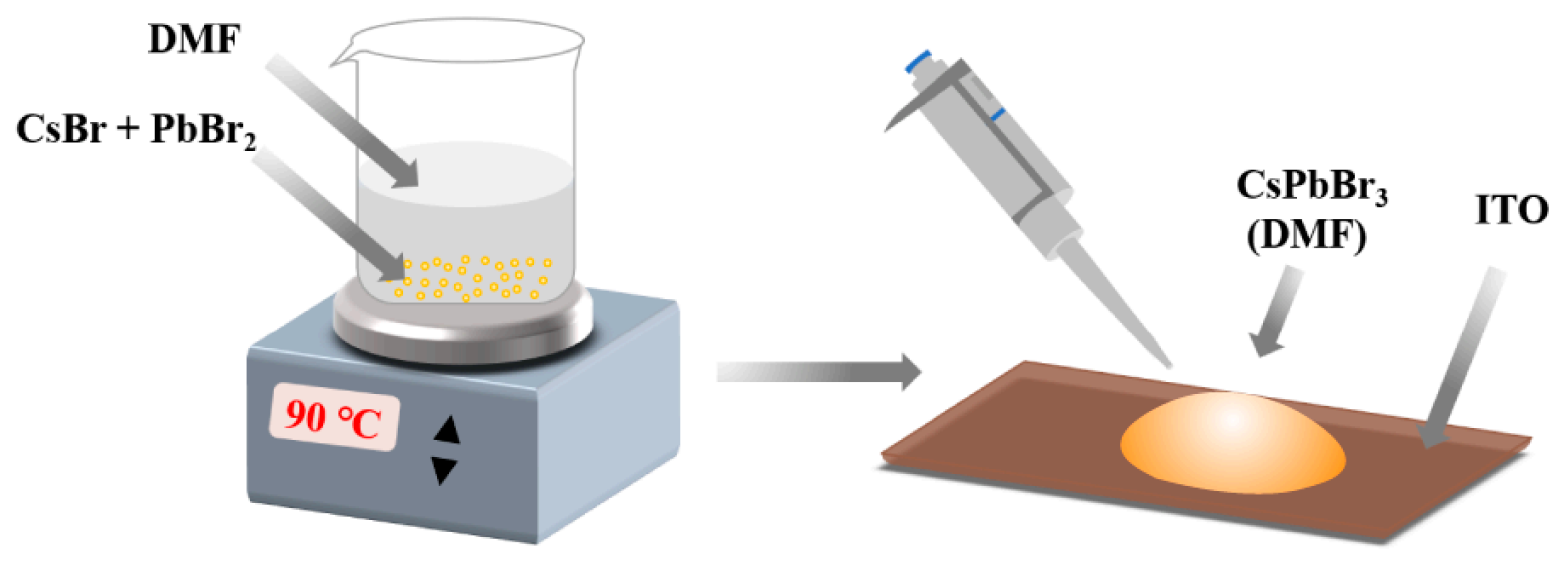
Basic process of liquid-phase synthesis of CsPbBr_3_ nanosheets.

The low-temperature liquid phase method was used to synthesize all the CsPbBr_3_ nanosheets in this study [18]. This approach serves as a promising alternative to chemical vapor deposition, enabling the growth of high-quality perovskite single crystals on arbitrary substrates while facilitating the development of perovskite-based devices. The synthesis was performed using the following high-purity reagents (Aladdin Co., Shanghai, China): cesium bromide (CsBr, 99.9%), lead bromide (PbBr_2_, 99.9%), and N, N-dimethylformamide (DMF, 99.5%). As shown in Figure 1, the sample preparation procedures were summarized as follows: 1 mmol CsBr and 2 mmol PbBr_2_ were mixed in a beaker with 3 mL DMF solution on a heating plate at 90 °C. Then, 20 μL of the mixture was pipetted onto the ITO substrate. As the temperature decreased, DMF also evaporated continuously, while part of the mixture was absorbed by the capillary, which accelerated the formation of CsPbBr_3_ nanosheets. After the entire evaporation of the DMF solution, high-quality CsPbBr_3_ nanosheets were obtained on the substrate.

### 2.2. Sample Characterization

The morphological characterization of the samples was performed using an Optical Microscope (OM, E200MW, Nikon, Shanghai, China), Scanning Electron Microscope (SEM, SU5000, Hitachi, Japan), and Atomic Force Microscope (AFM, Cypher S, Oxford Instruments, MA, USA). Crystalline phase analysis was conducted by X-ray diffractometer (XRD, Ultime IV, Rigaku, Japan) with Cu-Kα1 radiation, λ = 0.154 nm, employing a scanning angle range of 10–45°at a rate of 5°/min.

## 3. Results and Discussion

### 3.1. Sample Topography and Structure

**Figure 2:**
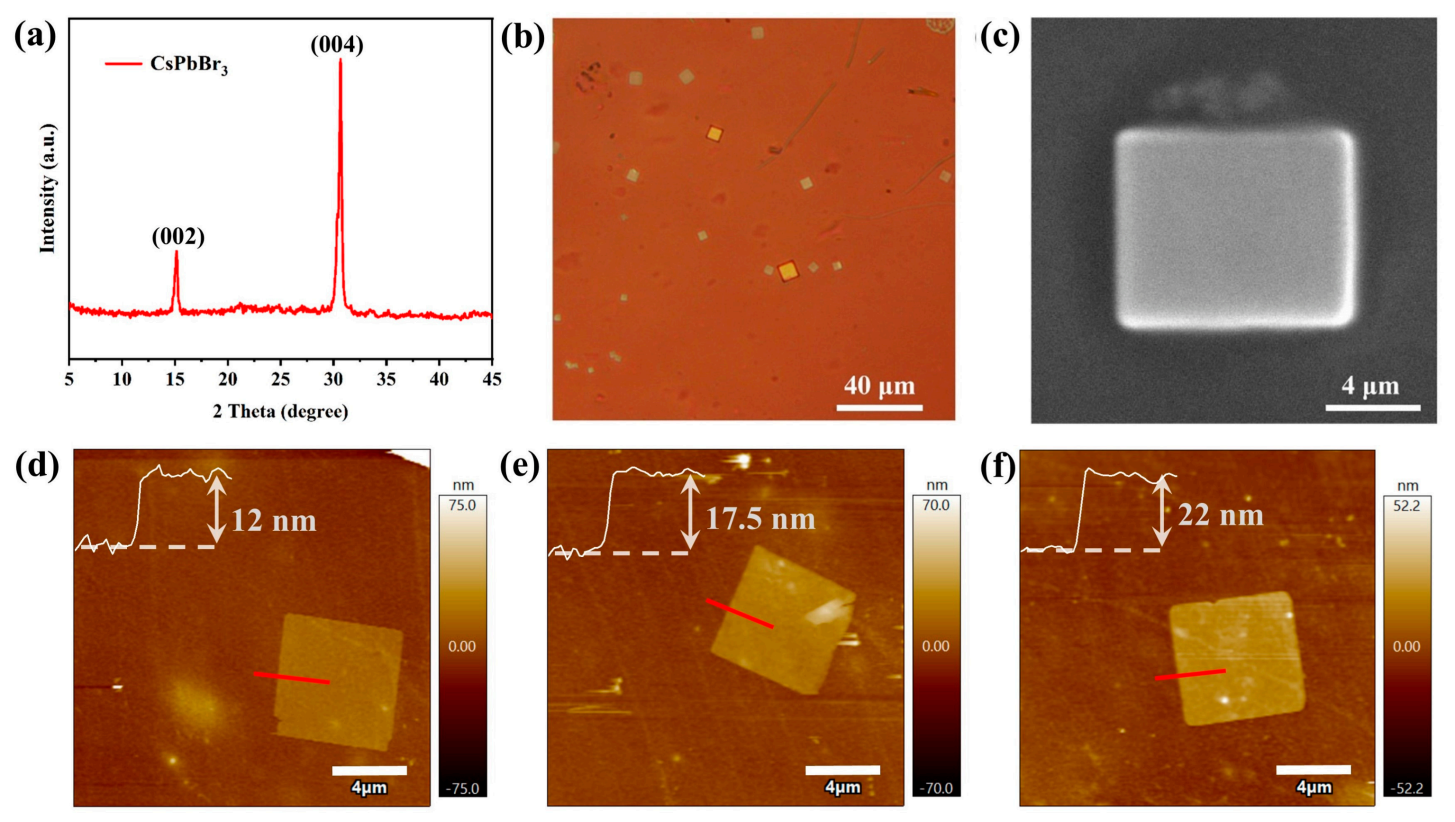
(**a**) XRD patterns of CsPbBr_3_ nanosheets. (**b**) Photomicrograph of CsPbBr_3_ on ITO substrate. (**c**) SEM image of CsPbBr_3_ on ITO substrate. (**d**–**f**) AFM morphology of CsPbBr_3_ nanosheets and the corresponding height profile marked by the red line.

The phase purity of the as-synthesized material has been confirmed by the X-ray diffraction (XRD) pattern, as shown in Figure 2a. The diffraction peaks in the XRD spectrum can be well indexed to the orthorhombic phase structure of the CsPbBr_3_ (JCPDS No. 54-0752, a = 8.21, b = 8.26, and c = 11.76 Å) [18,19]. It exhibits only the (002) and (004) diffraction peaks, indicating preferential crystallographic orientation along the (002) and (004) planes during growth. Figure 2b presents an optical micrograph of the CsPbBr_3_ nanosheet deposited on an ITO substrate. The image reveals a well-defined square morphology with minimal surface defects and a lateral dimension of approximately 10 μm. Notably, the contrast variation between the sample and substrate suggests non-uniform thickness distribution across the sample. Given the resolution limitations of optical microscopy for detailed surface characterization, we employed scanning electron microscopy (SEM) for higher-magnification analysis. As shown in Figure 2c, the SEM image demonstrates a smooth surface morphology with sharp, well-defined edges.

To determine the thickness of CsPbBr_3_ nanosheets, atomic force microscopy (AFM) was performed in tapping mode to minimize the tip-induced sample damage. The CsPbBr_3_ nanosheets were synthesized by a solution-phase method, where nucleation and crystallization occur during solvent evaporation. Due to inherent variations in evaporation rates, the resulting nanosheets exhibit non-uniform thicknesses. Based on optical contrast differences between the nanosheet and substrate, three representative regions were selected for AFM analysis. Height profiles extracted along the solid red lines in Figure 2d–f reveal nanosheet thicknesses of 12 nm, 17.5 nm, and 22 nm, which are nominal thicknesses of the three representative nanosheets selected for electrical characterization rather than statistical averages, demonstrating the thickness variability across CsPbBr_3_ nanosheets.

### 3.2. Electrical Properties of CsPbBr_3_ Nanosheets

**Figure 3:**
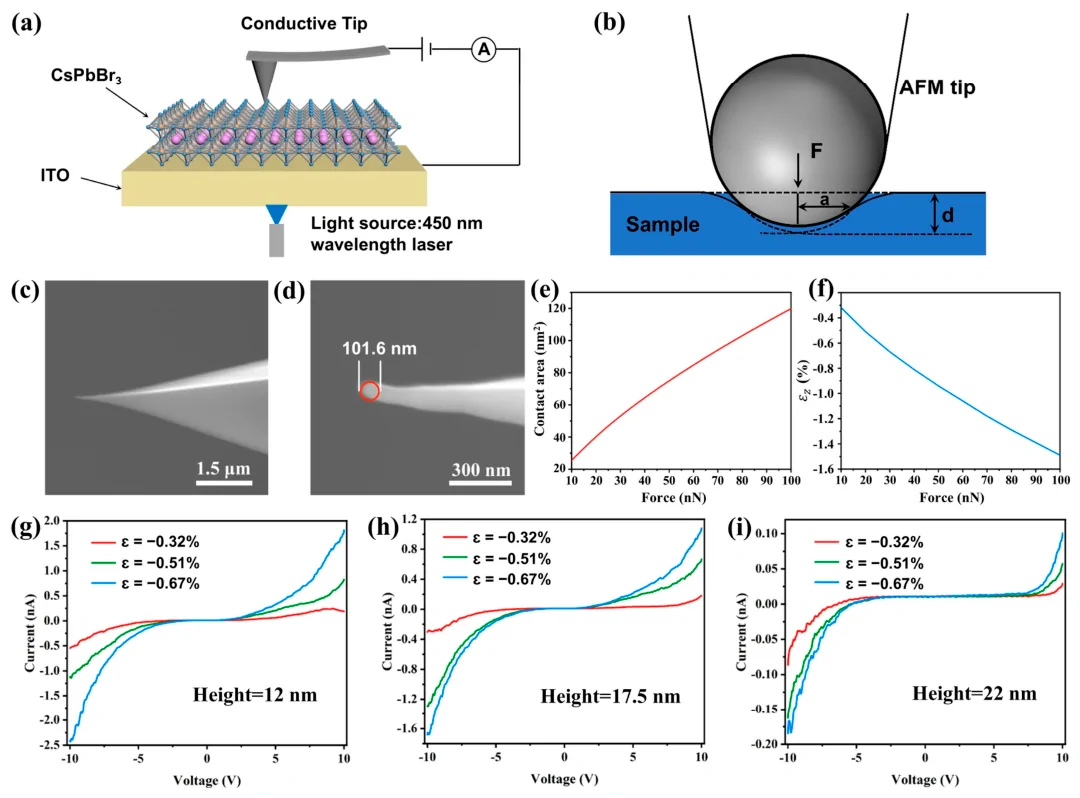
(**a**) Schematic diagram of the fabricated photodetector with the vertical structure under C-AFM measurement. (**b**) Contact model diagram of AFM tip and sample. SEM images of AFM tips: (**c**) initial, (**d**) after experiment. Relationship (**e**) between applied load and contact area and (**f**) between applied load and strain. I-V curves of samples with different thicknesses under strain. (**g**) 12 nm; (**h**) 17.5 nm; (**i**) 22 nm.

The current-voltage (I-V) characteristics of the CsPbBr_3_ nanosheet were measured under varying vertical pressures using conductive atomic force microscopy (C-AFM). C-AFM was used to produce the vertical structure photodetector of 2D CsPbBr_3_, as schematically depicted in Figure 3a. The semiconductor nanosheet of CsPbBr_3_ is sandwiched between the ITO substrate and a conductive Pt/Ir AFM tip (AC160TS-R3). The sample was illuminated with an external focusable laser of wavelength 450 nm (Fiber1, wyoptics, CHINA) to measure the photocurrent of this constructed device. In order to eliminate photo-thermal cantilever drift, the 450 nm laser was introduced from the bottom of the ITO substrate to irradiate the tip–sample junction, similar to the method reported previously [20]. The AFM tip–sample contact mechanics were modeled as shown in Figure 3b. The contact area under different loading conditions was quantified using Hertzian contact theory, which describes the relationship between applied load (*F*) and indentation depth (*d*) [21]:

(1) $$F=\frac{4}{3}E^{*}R^{1/2}d^{3/2}$$

where *R* is the radius of the AFM tip, and E^*^ is a correlation function of Young’s modulus, as well as the Poisson’s ratios:

(2) $$E^{*}={\left(\frac{1-{v_{T}}^{2}}{E_{T}}+\frac{1-{v_{F}}^{2}}{E_{F}}\right)}^{-1}$$

where *E_T_* and *E_F_* are the Young’s moduli of the sample and tip, respectively, *v_T_* and *v_F_* are the Poisson’s ratios of the sample and tip, respectively. The contact radius (*a*) versus the applied pressure (*F*) is given by:

(3) $$a={\left(\frac{3\mathrm{FR}}{4E^{*}}\right)}^{1/3}$$

Thus, the tip–sample contact area (*A_c_*) can be expressed as:

(4) $$A_{c}=\pi \left(a^{2}+d^{2}\right)=\pi \left\{{\left(\frac{3\mathrm{FR}}{4E^{*}}\right)}^{2/3}+\frac{1}{R^{2}}{\left(\frac{3\mathrm{FR}}{4E^{*}}\right)}^{4/3}\right\}$$

The AFM tip radius was characterized using scanning electron microscopy (SEM) before and after experiments. The conductive Pt/Ir-coated probe exhibited an initial radius of 25 ± 10 nm (Figure 3c). Due to tip wear, the radius increased to approximately 50 nm after the first measurement (Figure 3d) and then remained relatively constant at ~50 nm for all subsequent measurements. This behavior was further verified by SEM observations at three stages: before measurement, after the first measurement, and after the last measurement (Figure S1 and Table S1, Supplementary Materials). Since the measured radii (48.7, 50.8, and 51.0 nm) are all close to 50 nm, an approximate value of 50 nm was adopted for the subsequent calculations. Based on previous studies (*v_T_* = 0.38 [22], *E_T_* = 170 GPa [23], *v_F_* = 0.33, *E_F_* = 15.8 Gpa [24]) and Equation (4), the relationship between the applied load and contact area was calculated for the worn tip (*R* = 50 nm), as shown in Figure 3e. Furthermore, the strain exerted by the AFM tip on the CsPbBr_3_ nanosheet (*ε_z_*) can be derived from the tip–sample contact mechanics model [25]:

(5) $$\varepsilon_{z}=\ln\frac{\pi a^{2}}{S_{\mathrm{TM}}}=\ln\frac{1}{1+\frac{1}{R^{2}}{\left(\frac{3\mathrm{FR}}{4E^{*}}\right)}^{2/3}}$$

The strain–load relationship was calculated for a tip radius of 50 nm (Figure 3f), with negative strain values indicating compressive deformation of the sample.

The current–voltage (I-V) characteristics of ITO/CsPbBr_3_/tip structures were systematically investigated under varying mechanical loads. The device architecture comprised semiconducting CsPbBr_3_ nanosheets sandwiched between an ITO substrate and a conductive AFM tip, forming two distinct metal–semiconductor junctions. Due to the considerable thickness and high resistivity of the CsPbBr_3_ nanosheets, a bias voltage range of −10 to 10 V was employed. Applied loads (10 nN, 20 nN, and 30 nN) were determined through cantilever deflection measurements using the nominal spring constant (26 N/m), corresponding to compressive strains (*ε*) of −0.32%, −0.51%, and −0.67%, respectively. As summarized in Table S1 (Supplementary Materials), the tip radii measured by SEM (48.7, 50.8, and 51.0 nm) are all close to 50 nm. The use of an approximate radius of 50 nm therefore introduces only a minor difference in the calculated strain (within ~0.02%), confirming the reasonableness of this approximation. These values were selected to prevent sample damage while enabling strain-dependent characterization. Figure 3g–i present the I-V characteristics for 12 nm-, 17.5 nm-, and 22 nm-thick CsPbBr_3_ nanosheets under different loads. All samples exhibit pronounced rectification behavior and asymmetric I-V curves, suggesting differing Schottky barrier heights at the two interfaces. Current density–voltage (J–V) characteristics of CsPbBr_3_ nanosheets with different thicknesses under strain are provided in Figure S2 (Supplementary Materials). Notably, the electrical response demonstrates clear strain dependence: increased compressive strain enhances conductivity and reduces turn-on voltage. This phenomenon is consistently observed across all nanosheet thicknesses investigated.

Thicker CsPbBr_3_ nanosheets typically exhibit lower turn-on voltages under identical strain and bias conditions. The I-V characteristics of these Schottky junction devices can be described by thermionic emission theory. The current flow through the device *I_t_* follows:

(6) $$I_{t}=AA^{*}T^{2}\exp(-\frac{{\mathrm{q\varphi}}_{B}}{\mathrm{kT}})\exp(\frac{\mathrm{qV}}{\eta \mathrm{kT}})$$

where *A*, A^*^, *T*, *k*, *q*, η, *φ_B_*, *V* are the contact area, Richardson constant (100 A/cm^2^K^2^ for the CsPbBr_3_), temperature, Boltzmann constant, electron charge, ideality factor, Schottky barrier height, and applied voltage, respectively. To obtain the Schottky barrier for the device, we perform a simple deformation of the above equation to obtain [26]:

(7) $$\ln I_{t}=\ln\left(AA^{*}T^{2}\right)-\frac{{\mathrm{q\varphi}}_{B}}{\mathrm{kT}}+\frac{\mathrm{qV}}{\eta \mathrm{kT}}$$

**Figure 4:**
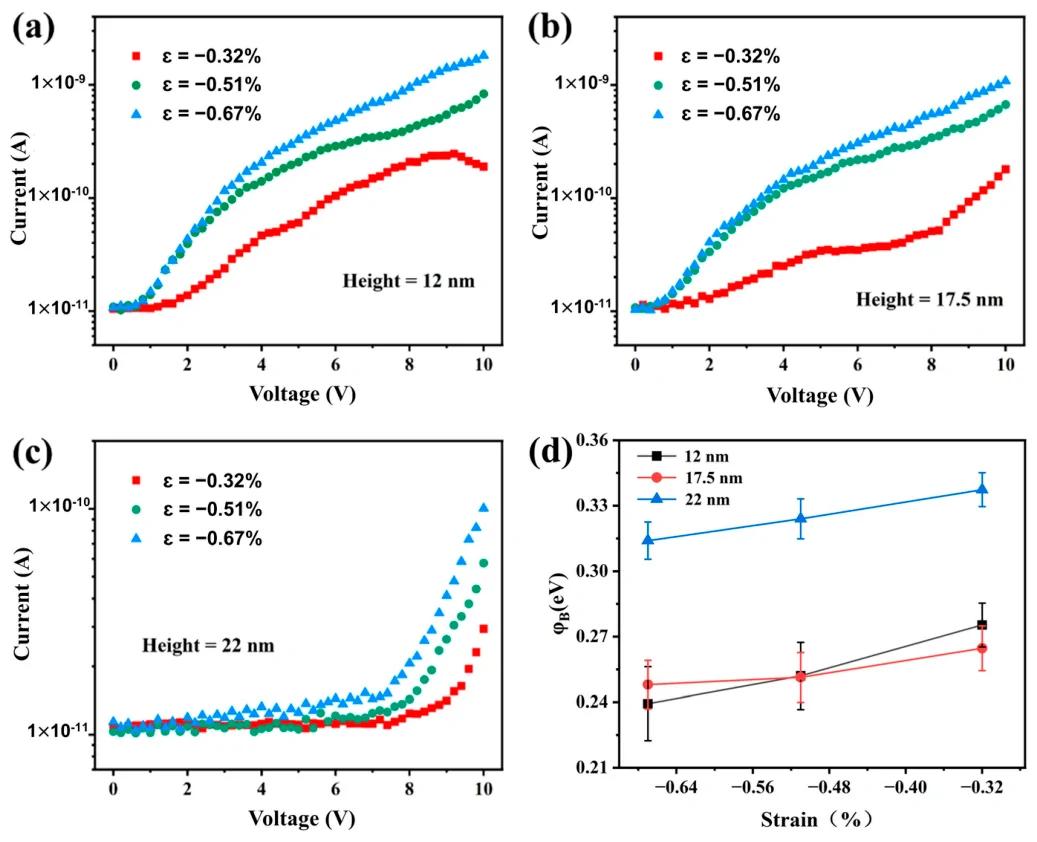
(**a**–**c**) Positive half logarithm current voltage curve. (**d**) Barrier versus strain curve for samples with different thicknesses.

Based on the above relationship, the forward-bias characteristics *lnI* − *V* curve was plotted as shown in Figure 4. Linear regression analysis of these curves yielded the Schottky barrier heights for CsPbBr_3_ nanosheets of varying thicknesses under different strain conditions (Figure 4d). The error bars in Figure 4d represent the uncertainty in the extracted barrier heights, and the corresponding statistical data are summarized in Table S2 (Supplementary Materials). Two key trends emerged: (1) at constant strain, thicker samples exhibited higher barrier heights (12 nm: 0.275 eV, 0.252 eV, and 0.239 eV; 17.5 nm:0.265 eV, 0.251 eV, and 0.248 eV; 22 nm:0.337 eV, 0.324 eV, and 0.314 eV), and (2) for a given thickness, increasing the compressive strain (−0.32%, −0.51%, and −0.67%) systematically reduced the barrier height by 36 meV, 17 meV, and 23 meV for 12 nm, 17.5 nm, and 22 nm nanosheets, respectively. This strain-dependent modulation of the tip–sample contact barrier directly influences the device’s electrical characteristics.

**Figure 5:**
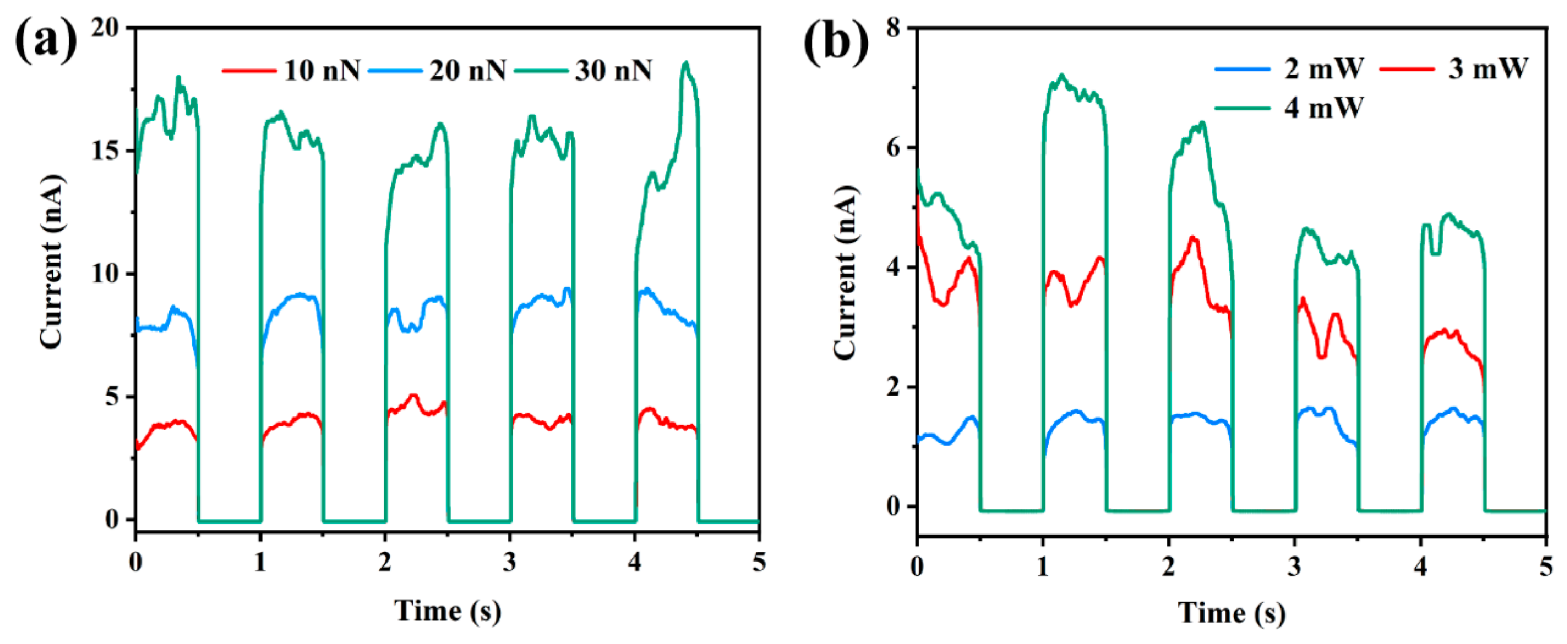
(**a**) Current–time curve under different loads. (**b**) Current–time curve under different optical power.

Localized strain engineering via AFM tip compression reduces the band gap of CsPbBr_3_ nanosheets, enhancing both photogenerated carrier density and recombination rates, thereby improving optoelectronic performance. Electrical characterization of the heterojunction revealed superior response in 12 nm-thick nanosheets, prompting detailed photoelectric measurements under varying strain conditions. Given the small sample dimensions (about 10 μm × 10 μm), photocurrent measurements required combined optical excitation (450 nm laser) with an applied 10 V square-wave bias (1 Hz) to achieve detectable signals. To probe the effect of applied force on the photoresponse, current–time (I-t) curves were performed simultaneously under different C-AFM tip loading force from 10 nN to 30 nN. Figure 5a demonstrates the strain-dependent photoresponse, showing a remarkable increase from 3.5 nA to 16 nA as the compressive load rises from 10 nN to 30 nN. Corresponding power-dependent measurements (Figure 5b) reveal photocurrent enhancement from 1 nA to 7 nA with increasing laser power (2 mW to 4 mW) at a fixed 10 nN load. These results conclusively demonstrate that localized strain engineering significantly enhances the optoelectronic performance of 2D CsPbBr_3_ nanosheets.

**Figure 6:**
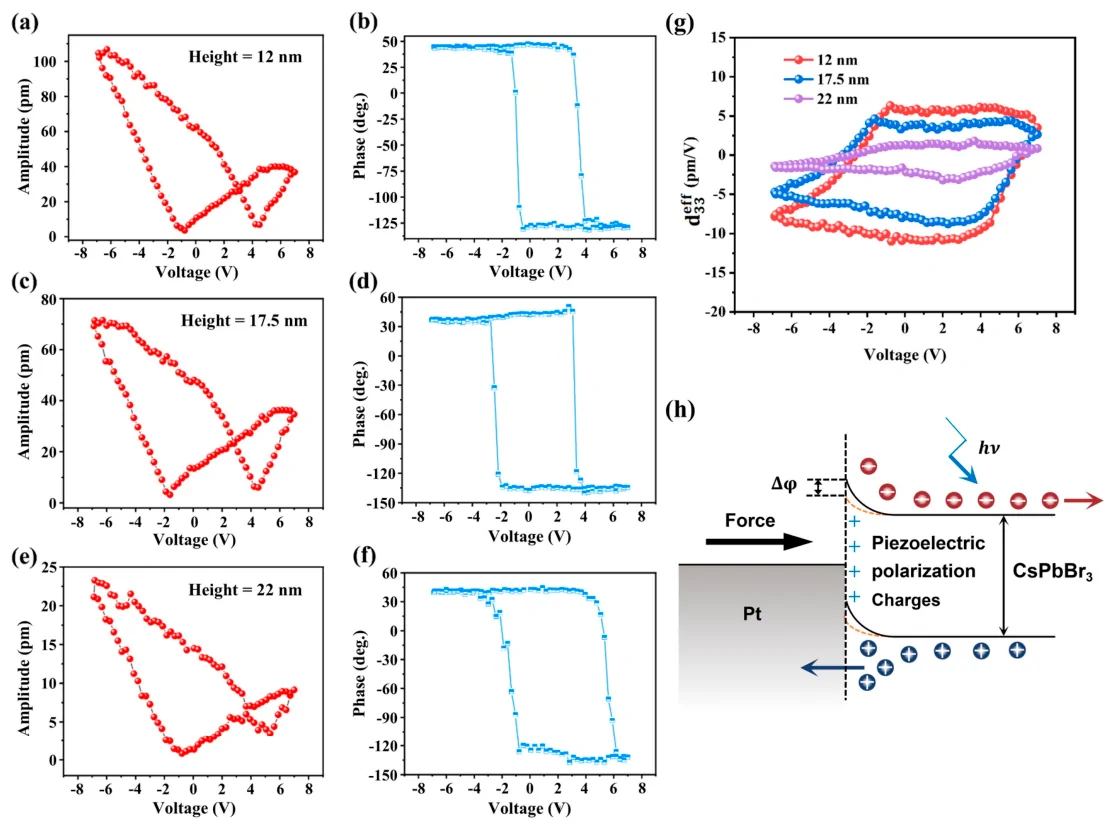
Piezoelectric amplitude (**a**,**c**,**e**) and phase curve (**b**,**d**,**f**) of CsPbBr_3_ nanosheets with different thicknesses. (**g**) $d_{33}^{eff}$-V curves of CsPbBr_3_ nanosheets with different thicknesses. (**h**) The energy band diagram of the piezotronics effect on the CsPbBr_3_ nanosheet.

The piezoelectric effect represents a fundamental electromechanical coupling phenomenon. Our previous current–voltage (I-V) characterization and photoelectric measurements of CsPbBr_3_ nanosheets under varying strain conditions demonstrated this coupling behavior [27]. To quantitatively assess the piezoelectric properties, we performed piezoelectric force microscopy (PFM) on CsPbBr_3_ nanosheets of different thicknesses (12 nm, 17.5 nm, and 22 nm). During PFM measurements, an AC bias voltage (−7 V to 7 V) was applied across the sample. Figure 6a,c,e display the amplitude responses of CsPbBr_3_, revealing typical butterfly loops with maximum amplitudes of 110 pm, 72 pm, and 23 pm at −7 V for 12 nm, 17.5 nm, and 22 nm nanosheets, respectively. The observed asymmetry in these loops correlates with the Schottky barrier differences at the electrodes, consistent with our earlier I-V measurements. Corresponding phase images (Figure 6b,d,f) exhibit uniform 180° domain switching for the different thicknesses of CsPbBr_3_ nanosheets, indicating a high-quality crystalline structure with minimal defects. The clear domain wall motion under applied voltage further confirms the strong piezoelectric response in these nanosheets.

Each data point in the butterfly curve corresponds to the piezoelectric displacement at a specific driving voltage. From the amplitude response, the effective vertical piezoelectric coefficient ($d_{33}^{eff}$) of the CsPbBr_3_ nanosheet can be quantitatively determined using the constitutive piezoelectric equation [28]:

(8) $$d_{33}^{\mathrm{eff}}=\frac{D-D_{1}}{V-V_{1}}$$

where *D* is the measured value of piezoelectric deformation, *V* is the applied voltage, *D*_1_ is the piezoelectric displacement at the intersection of the curves, and *V*_1_ is the loaded voltage at the intersection. The above equation calculates the curve of $d_{33}^{eff}$ versus V, as shown in Figure 6g. The measured $d_{33}^{eff}$ values may be systematically overestimated due to several experimental factors: (1) non-ideal tip–sample alignment during PFM characterization and (2) potential contributions from electrostrictive effects to the butterfly curve response. Consequently, the calculated piezoelectric coefficients typically exceed the intrinsic values of the material. Nevertheless, the relative magnitude of $d_{33}^{eff}$ provides a meaningful comparison of piezoelectric properties across samples. The two branches of the butterfly curves are asymmetric, so the values extracted at different bias voltages show a certain spread (Table S3, Supplementary Materials). The absolute values of $d_{33}^{eff}$ should therefore be interpreted with care, whereas the relative thickness-dependent trend is more reliable and is used to support the conclusions of this work. The calculated $d_{33}^{eff}$ values for 12 nm, 17.5 nm, and 22 nm CsPbBr_3_ nanosheets were 5.8 pm/V, 3.7 pm/V, and 1.2 pm/V, respectively, demonstrating an inverse relationship between piezoelectric response and nanosheet thickness. This thickness dependence correlates well with the observed electrical response trends in previous measurements.

Piezoelectric semiconductors are typically characterized using the piezotronic effect. According to fundamental piezotronic principles, the polarization vector *P* relates to strain (*S*)*_jk_* under small, uniform deformations, as in [29]:

(*P*)*_i_* = (*e*)*_ijk_*(*S*)*_jk_* (9)

where (*e*)*_ijk_* represents the third-rank piezoelectric tensor. For uniaxial strain along the crystallographic c-axis, the induced piezoelectric polarization simplifies to:

*P_z_* = *e*_33_*s*_33_ = *qρ_piezo_W_piezo_* (10)

where *q* is the electron charge, *ρ_piezo_* is the polarization charge density, and *W_piezo_* is the piezoelectric charge width. The strain-induced piezoelectric effect modifies the Fermi energy level (*E_F_*) according to:

(11) $$E_{F}=E_{0}-\frac{q^{2}\rho_{\mathrm{piezo}}W_{\mathrm{piezo}}^{2}}{2\varepsilon_{s}}=E_{0}-\frac{e_{33}s_{33}qW_{\mathrm{piezo}}}{2\varepsilon_{s}}$$

where *E*_0_ represents the initial Fermi energy level of the sample, and *ε_s_* denotes the dielectric constant of the sample. From the above equation, it can be found that, if the sample is subjected to compressive strain in the vertical direction (*e* < 0), then it induces a negative shift in the Fermi energy level brought about by the piezoelectric effect, resulting in an upward shift of the total Fermi energy level. The rise in the Fermi energy level reduces the effective Schottky barrier height, which results in an enhancement of the electrical and optoelectronic properties of the sample.

## 4. Conclusions

This study focuses on the strain-modulated electrical transport of inorganic CsPbBr_3_ nanosheets, a system in which strain engineering provides a route to tuning carrier transport through the piezotronic effect. A systematic investigation of the electrical properties yields the following key insights:

1. CsPbBr_3_ nanosheets with controlled thickness variations were successfully synthesized on ITO substrates via a low-temperature solution-phase method. Structural analyses confirmed high surface quality and orthorhombic crystallinity, as evidenced by XRD;
2. Strain-modulated electrical behavior: A tip/CsPbBr_3_/ITO vertical heterostructure was fabricated using C-AFM. Current–voltage (I-V) characterization under compressive strain revealed strain-dependent rectification behavior, with a marked enhancement in charge transport properties at higher strain levels;
3. Mechanistic origins of barrier modulation: Experimental I-V characteristics were correlated with the thermionic emission model, demonstrating a compressive-strain-induced reduction in Schottky barrier height. Piezoresponse force microscopy (PFM) quantified the vertical piezoelectric coefficients ($d_{33}^{eff}$) as 5.8 pm/V, 3.7 pm/V, and 1.2 pm/V for 12 nm, 17.5 nm, and 22 nm nanosheets, respectively. Consistent with piezotronic principles, the barrier lowering is mechanistically explained by piezoelectric polarization-induced band structure modification.

Nevertheless, the PFM-derived $d_{33}^{eff}$ values may be overestimated due to non-ideal tip–sample alignment and possible electrostrictive contributions, and the tip-induced strain is highly localized and non-uniform. The study is also limited to three thicknesses and to individual nanosheets rather than integrated devices. Future work should therefore decouple piezoelectric and electrostrictive contributions, extend the approach to other perovskite compositions, and integrate the strained nanosheets into practical optoelectronic devices.

Overall, this work provides a quantitative correlation between local tip-induced strain, thickness-dependent piezoelectricity, and Schottky barrier modulation in vertical CsPbBr_3_ nanosheet junctions, offering a piezotronics–thermionic-emission framework for strain-engineered perovskite optoelectronics.

## Data Availability

All the data supporting the findings of this study are available within this article and its Supplementary Materials files. All raw images and source data are available from the authors upon reasonable request.

## Supplementary Materials

The following supporting information can be downloaded at https://www.mdpi.com/article/10.3390/nano16201284/s1: Figure S1: SEM images of AFM tips: (a) initial. (b) after the first measurement, and (c) after the last measurement.; Table S1: Measured and approximate AFM tip radius and the corresponding calculated strain under different applied loads; Figure S2: Current density–voltage (J–V) characteristics of CsPbBr_3_ nanosheets with different thicknesses under strain; Table S2: Statistical data of the extracted Schottky barrier heights (φB) for CsPbBr_3_ nanosheets of different thicknesses under different compressive strains; Table S3: Effective $d_{33}^{\mathrm{eff}}$ values (|$d_{33}^{\mathrm{eff}}$|, pm/V) of CsPbBr_3_ nanosheets at ±4, ±5 and ±7 V, calculated from the digitized butterfly curves in Figure 6a,c,e using $d_{33}^{\mathrm{eff}}$ = (Z − Zc)/(V − Vc).

## References

[1] Prete P., Lovergine N. (2025). High efficiency III-V nanowire solar cells: The road ahead. Nano Futur..

[2] Chen G., Sun G., Ding Y.J., Prete P., Miccoli I., Lovergine N., Shtrikman H., Kung P., Livneh T., Spanier J.E. (2013). Direct Measurement of Band Edge Discontinuity in Individual Core–Shell Nanowires by Photocurrent Spectroscopy. Nano Lett..

[3] Assirey E.A.R. (2019). Perovskite synthesis, properties and their related biochemical and industrial application. Saudi Pharm. J..

[4] Green M.A., Ho-Baillie A., Snaith H.J. (2014). The emergence of perovskite solar cells. Nat. Photon..

[5] Wehrenfennig C., Eperon G.E., Johnston M.B., Snaith H.J., Herz L.M. (2014). High charge carrier mobilities and lifetimes in organolead trihalide perovskites. Adv. Mater..

[6] Saidaminov M.I., Abdelhady A.L., Murali B., Alarousu E., Burlakov V.M., Peng W., Dursun I., Wang L., He Y., Maculan G. (2015). High-quality bulk hybrid perovskite single crystals within minutes by inverse temperature crystallization. Nat. Commun..

[7] Gao Y., Wu Y., Liu Y., Lu M., Yang L., Wang Y., Yu W.W., Bai X., Zhang Y., Dai Q. (2020). Interface and grain boundary passivation for efficient and stable perovskite solar cells: The effect of terminal groups in hydrophobic fused benzothiadiazole-based organic semiconductors. Nanoscale Horiz..

[8] Zuo C., Bolink H., Han H., Huang J., Cahen D., Ding L. (2016). Advances in perovskite solar cells. Adv. Sci..

[9] Lee J.-W., Choi Y.J., Yang J.-M., Ham S., Jeon S.K., Lee J.Y., Song Y.-H., Ji E.K., Yoon D.-H., Seo S. (2017). In-situ formed type I nanocrystalline perovskite film for highly efficient light-emitting diode. ACS Nano.

[10] Xu J., Zhou Y., Jiang H., Zhu K., Wan Y., Lai M., Xu S. (2025). Flexible and Stable Piezoelectric Nanogenerators Based on Monoclinic Phase CsPbBr_3_ Perovskite Nanocrystals. Nanoscale.

[11] Cao F., Meng L., Wang M., Tian W., Li L. (2019). Gradient energy band driven high-performance self-powered perovskite/CdS photodetector. Adv. Mater..

[12] Premkumar S., Kundu K., Umapathy S. (2019). Impact of cesium in methylammonium lead bromide perovskites: Insights into the microstructures, stability and photophysical properties. Nanoscale.

[13] Ding J., Du S., Zhou T., Yuan Y., Cheng X., Jing L., Yao Q., Zhang J., He Q., Cui H. (2019). Cesium Decreases Defect Density and Enhances Optoelectronic Properties of Mixed MA_1–x_Cs_x_PbBr_3_ Single Crystal. J. Phys. Chem. C.

[14] Li F., Wang H., Chen Z., Liu X., Wang P., Zhang W., Dong H., Fu J., Wang Z., Shao Y. (2024). Aging CsPbBr_3_ Nanocrystal Wafer for Ultralow Ionic Migration and Environmental Stability for Direct X-ray Detection. ACS Appl. Mater. Interfaces.

[15] Liang X., Ren X., Yang S., Liu L., Xiong W., Cheng L., Ma T., Liu A. (2021). Theoretical study of the influence of doped niobium on the electronic properties of CsPbBr_3_. Nanoscale Adv..

[16] Xu Q., Yang Z., Peng D., Xi J., Lin P., Cheng Y., Liu K., Pan C. (2019). WS_2_/CsPbBr_3_ van der Waals heterostructure planar photodetectors with ultrahigh on/off ratio and piezo-phototronic effect-induced strain-gated characteristics. Nano Energy.

[17] Li X., Meng Y., Fan R., Fan S., Dang C., Feng X., Ho J.C., Lu Y. (2021). High elasticity of CsPbBr_3_ perovskite nanowires for flexible electronics. Nano Res..

[18] Zheng W., Xiong X., Lin R., Zhang Z., Xu C., Huang F. (2018). Balanced photodetection in one-step liquid-phase-synthesized CsPbBr_3_ micro-/nanoflake single crystals. ACS Appl. Mater. Interfaces.

[19] Rodová M., Brožek J., Knížek K., Nitsch K. (2003). Phase transitions in ternary caesium lead bromide. J. Therm. Anal. Calorim..

[20] Pathak C.S. (2025). Mapping the photocurrent and photovoltage of hybrid perovskite thin films under concentrated sunlight. Macromol. Res..

[21] Johnson K.L. (1987). Contact Mechanics.

[22] Siegrist M.E., Löffler J.F. (2007). Bulk metallic glass–graphite composites. Scr. Mater..

[23] Venkataraman S., Kohlstedt D.L., Gerberich W.W. (1992). Microscratch analysis of the work of adhesion for Pt thin films on NiO. J. Mater. Res..

[24] Rakita Y., Cohen S.R., Kedem N.K., Hodes G., Cahen D. (2015). Mechanical Properties of APbX_3_ (A = Cs or CH_3_NH_3_; X = I or Br) Perovskite Single Crystals. MRS Commun..

[25] Ahn J.H., Kwon D. (2001). Derivation of plastic stress–strain relationship from ball indentations: Examination of strain definition and pileup effect. J. Mater. Res..

[26] Cui D., Tian C., Wang Y., Wang F., Yang Z., Mei J., Liu H., Zhao D. (2019). Back-to-back Schottky junction photodetectors based on CVD grown CsPbBr_3_ microcrystalline striped films. AIP Adv..

[27] Wei Y., Zheng X., Chu L., Dong H. (2022). Piezo-Phototronic Enhancement of Vertical Structure Photodetectors Based on 2D CsPbBr_3_ Nanosheets. J. Nanoelectron. Optoelectron..

[28] Wu D.-X., Cheng H.-B., Zheng X.-J., Wang X.-Y., Wang D., Li J. (2015). Fabrication and piezoelectric characterization of single crystalline GaN nanobelts. Chin. Phys. Lett..

[29] Zhang Y., Liu Y., Wang Z.L. (2011). Fundamental theory of piezotronics. Adv. Mater..

