# Supplementary material for: Electrical Properties of Inorganic Perovskites CsPbBr3 Nanosheet Based on Strain Engineering
Source: Nanomaterials (Basel). 2026 Oct 10;16(20):1284. doi: 10.3390/nano16201284 (PMC13707719; doi:10.3390/nano16201284)
Supplement: Supplementary file 1 [file nanomaterials-16-01284-s001.zip › nanomaterials-4582196-supplementary.pdf]

Supplementary Materials

Electrical properties of inorganic perovskites $CsPbBr_3$ nanosheet based on strain engineering

Yi Wu [1], Ruixue Wang [1] and Hui Dong [1,*]

[1] State Key Laboratory of Precision Electronic Manufacturing Technology and Equipment, School of Electromechanical Engineering, Guangdong University of Technology, Guangzhou 510006, PR China

* Correspondence: huidong@xtu.edu.cn (H.D.)

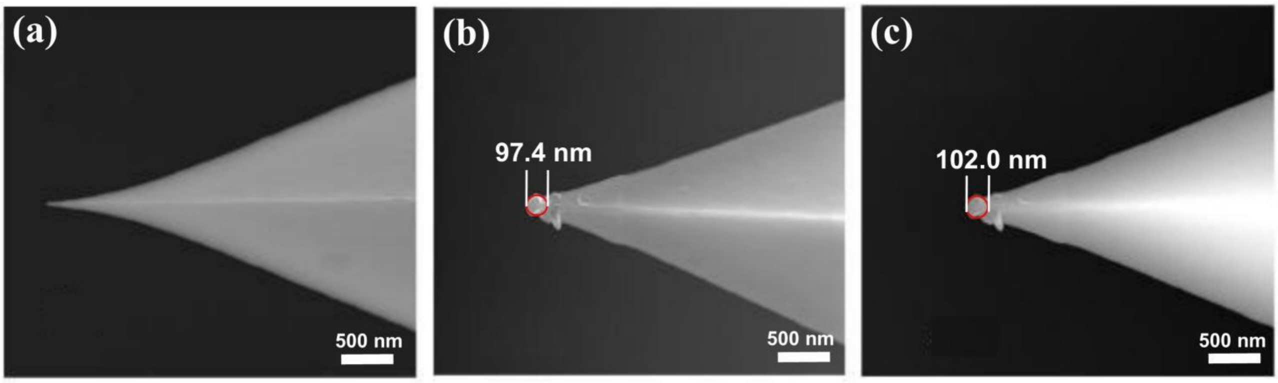

Figure S1. SEM images of AFM tips: (a) initial. (b) after the first measurement, and (c) after the last measurement.

Table S1. Measured and approximate AFM tip radius and the corresponding calculated strain under different applied loads.

AFM tip radius (R)/nm	Loads /nN	Strain(ε_z)
48.7 (Fig. S1b)	10	-0.33%
	20	-0.53%
	30	-0.69%
50.8 (Fig. 3d)	10	-0.32%
	20	-0.50%
	30	-0.66%
51.0 (Fig. S1b)	10	-0.31%
	20	-0.50%
	30	-0.65%
50 (Approximate value)	10	-0.32%
	20	-0.51%
	30	-0.67%

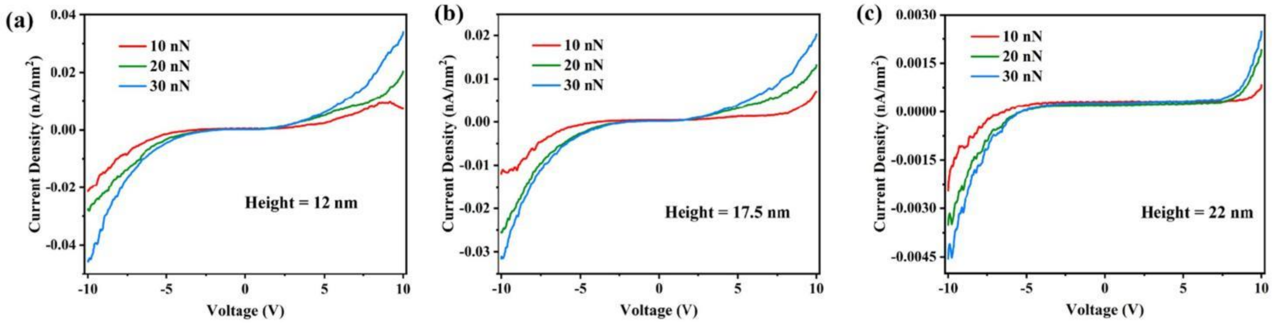

Figure S2. Current density–voltage (J–V) characteristics of $CsPbBr_3$ nanosheets with different thicknesses under strain. (a) 12 nm; (b) 17.5 nm; (c) 22 nm.

Table S2. Statistical data of the extracted Schottky barrier heights (φB) for $CsPbBr_3$ nanosheets of different thicknesses under different compressive strains. (Corresponding to the error bars in Figure 4d.)

Thickness / nm	Load / nN	Strain / （ε_z）	φ_B (5 V) / eV	φ_B (8 V) / eV	φ_B (10 V) / eV	$\overline{\varphi_B}$ ± σ / eV
12	10	-0.32%	0.286	0.274	0.266	0.275 ± 0.010
	20	-0.51%	0.269	0.248	0.239	0.252 ± 0.015
	30	-0.67%	0.258	0.235	0.225	0.239 ± 0.017
17.5	10	-0.32%	0.276	0.262	0.256	0.265 ± 0.010
	20	-0.51%	0.264	0.248	0.242	0.251 ± 0.011
	30	-0.67%	0.260	0.246	0.238	0.248 ± 0.011
22	10	-0.32%	0.346	0.335	0.331	0.337 ± 0.008
	20	-0.51%	0.334	0.322	0.316	0.324 ± 0.009
	30	-0.67%	0.323	0.313	0.306	0.314 ± 0.009

Table S3. Effective d_{33}^{eff} values ($|d_{33}^{eff}|$, pm/V) of $CsPbBr_3$ nanosheets at ±4, ±5 and ±7 V, calculated from the digitized butterfly curves in Fig. 6(a, c, e) using $d_{33}^{eff} = (Z - Zc)/(V - Vc)$.

Thickness (nm)	d_{33}^{eff} (+7 V)	d_{33}^{eff} (+5 V)	d_{33}^{eff} (+4 V)	d_{33}^{eff} (−4 V)	d_{33}^{eff} (−5 V)	d_{33}^{eff} (−7 V)	$\overline{d_{33}^{eff}} \pm \sigma$
12	2.44	5.37	12.48	6.62	7.54	7.71	7.03±3.30
17.5	2.92	4.49	8.39	3.94	4.57	4.89	4.87±1.86
22	0.62	1.21	1.45	1.20	1.27	1.48	1.21±0.31

Note: Values are $|d_{33}^{eff}|$ averaged over the two scanning branches, where $d_{33}^{eff} = (Z - Zc)/(V - Vc)$, Z is the digitized piezoresponse amplitude and (Vc, Zc) is the intersection of the two branches, being (+2.97 V, 26.2 pm), (+2.76 V, 22.6 pm) and (+3.31 V, 6.8 pm) for 12, 17.5 and 22 nm, respectively.
